# Supplementary material for: Putting people in context: ERP responses to bodies in natural scenes
Source: PLoS One. 2023 Oct 26;18(10):e0283673. doi: 10.1371/journal.pone.0283673 (PMC10602242; doi:10.1371/journal.pone.0283673)
Supplement: Attachment — Submitted filename: BalasNudnou_PloS_Response.R2.docx [file pone.0283673.s002.docx]

**Response to Reviewers**

We would once again like to thank our reviewers for their comments on the most recent draft of our manuscript. We were pleased to see that they felt we had adequately addressed their concerns and we have made additional changes to the text (specifically the figures) to respond to their most recent suggestions. We reproduce their comments in full below, with our responses in **bold.**

Review Comments to the Author

Reviewer #1: The authors have addressed most of my concerns. However, it seems like the example stimuli figures have been removed in this revision. If at all possible, I would strongly encourage the authors to bring these back in (if the experimental stimuli are not allowed to be reprinted, the authors could create new stimuli that are representative of the experimental stimuli). Seeing the example stimuli (both upright and inverted) for both experiments really helps to understand the manipulation in a single glance.

**Thank you for this suggestion. We have created new images ourselves (that we retain copyright to) that are now included to illustrate the upright and inverted stimulus conditions in both experiments.**

Reviewer #2: I thank the Authors for better characterising the research question specifying that inversion served as a control for body processing specificity. Generally, I think the paper has improved with the revisions and the conclusions taken are supported by the data.

**Thank you for these comments.**

Minor:

Figure 3a 3b - 10a 10b

Please add labels at the axis (electrode n. and timepoint).

**We have added these as requested in the revised figures.**

Experiment 2: N190 peak latency analysis - You report a marginal effect, please clarify whether this goes in the same direction of exp. 1 - Seems that it is the case from what I read in the discussions.

**We have made this explicit in the revised text.**

I would include inverted stimuli examples in the figures for completeness.

**We have done so.**

**Thank you again to both of our reviewers for their commentary on the revised draft.**
